# Supplementary material for: Multiple pairs of allelic MLA immune receptor-powdery mildew AVRA effectors argue for a direct recognition mechanism
Source: eLife. 2019 Feb 19;8:e44471. doi: 10.7554/eLife.44471 (PMC6414202; doi:10.7554/eLife.44471)
Supplement: Transparent reporting form [file elife-44471-transrepform.pdf]

eLIFE
1st Floor
24 Hills Road
Cambridge CB2 1JP, UK
P 01223 855340
W elifesciences.org
T @elife

# *eLife's* transparent reporting form

We encourage authors to provide detailed information *within their submission* to facilitate the interpretation and replication of experiments. Authors can upload supporting documentation to indicate the use of appropriate reporting guidelines for health-related research (see EQUATOR Network), life science research (see the BioSharing Information Resource), or the ARRIVE guidelines for reporting work involving animal research. Where applicable, authors should refer to any relevant reporting standards documents in this form.

If you have any questions, please consult our Journal Policies and/or contact us: editorial@elifesciences.org.

**Sample-size estimation**

- You should state whether an appropriate sample size was computed when the study was being designed
- You should state the statistical method of sample size computation and any required assumptions
- If no explicit power analysis was used, you should describe how you decided what sample (replicate) size (number) to use

Please outline where this information can be found within the submission (e.g., sections or figure legends), or explain why this information doesn't apply to your submission:

All molecular and biochemical experiments were performed at least three independent times (independent biological replicates with independently grown plants on different days). In case of moderate signal/noise ratios within biological replicates (Fig 4C) each experiment was additionally repeated at least twice completely independently by a second independent researcher and all data points obtained by all executing researches are displayed. Additionally, Y2H data (Fig 5) was also performedindependently by a second independent researcher.

**Replicates**

- You should report how often each experiment was performed
- You should include a definition of biological versus technical replication
- The data obtained should be provided and sufficient information should be provided to indicate the number of independent biological and/or technical replicates
- If you encountered any outliers, you should describe how these were handled
- Criteria for exclusion/inclusion of data should be clearly stated
- High-throughput sequence data should be uploaded before submission, with a private link for reviewers provided (these are available from both GEO and ArrayExpress)

Please outline where this information can be found within the submission (e.g., sections or figure legends), or explain why this information doesn't apply to your submission:

eLife Sciences Publications, Ltd is a limited liability non-profit non-stock corporation incorporated in the State of Delaware, USA, with company number 5030732, and is registered in the UK with company number FC030576 and branch number BR015634 at the address 1st Floor, 24 Hills Road, Cambridge CB2 1JP | August 2014

| |
|---|
| p values and minimum number of biological replicates are indicated in respective figure legends,<br>outlier points are displayed and included for statistical analysis to avoid biased data correction,<br>RNAseq data was submitted to GEO (accession no. 688 GSE110266).<br>Improved *B. graminis f.sp. hordei* isolate K1 assembly is deposited at EBI-ENA (accession number PRJEB30373). |

**Statistical reporting**

- Statistical analysis methods should be described and justified
- Raw data should be presented in figures whenever informative to do so (typically when N per group is less than 10)
- For each experiment, you should identify the statistical tests used, exact values of N, definitions of center, methods of multiple test correction, and dispersion and precision measures (e.g., mean, median, SD, SEM, confidence intervals; and, for the major substantive results, a measure of effect size (e.g., Pearson's r, Cohen's d)
- Report exact p-values wherever possible alongside the summary statistics and 95% confidence intervals. These should be reported for all key questions and not only when the p-value is less than 0.05.

Please outline where this information can be found within the submission (e.g., sections or figure legends), or explain why this information doesn't apply to your submission:

Statistical tests:
Genetic association analysis: Fisher's exact test described in Methods section, p values indicated in text ('Combined TWAS and Bgh genome analysis identified candidates for AVRa7, AVRa9, AVRa10, and AVRa22'),
Fig. 4 A-C, Fig.4-figure supplements 1 and 2 (parametric) ANOVA followed by Tukey's post hoc test, Fig. 5 A-C (non-parametric): Kruskal Wallis followed by Dunn test.
p values and minimum n indicated in respective figure legends.
Raw data: bar graphs and dot plots include all obtained data points for respective experiments.

(For large datasets, or papers with a very large number of statistical tests, you may upload a single table file with tests, Ns, etc., with reference to sections in the manuscript.)

**Group allocation**

- Indicate how samples were allocated into experimental groups (in the case of clinical studies, please specify allocation to treatment method); if randomization was used, please also state if restricted randomization was applied
- Indicate if masking was used during group allocation, data collection and/or data analysis

Please outline where this information can be found within the submission (e.g., sections or figure legends), or explain why this information doesn't apply to your submission:

Genetic association analysis grouping: association of transcript variation with observed avirulence phenotypes of Bgh isolates per barley MLA Near isogenic line, indicated in Method section and text ('Combined TWAS and Bgh genome analysis identified candidates for AVRa7, AVRa9, AVRa10, and AVRa22'),
Fig. 4 A-C grouping: per MLA, indicated in Figure legend.

**Additional data files ("source data")**

- We encourage you to upload relevant additional data files, such as numerical data that are represented as a graph in a figure, or as a summary table
- Where provided, these should be in the most useful format, and they can be uploaded as "Source data" files linked to a main figure or table
- Include model definition files including the full list of parameters used
- Include code used for data analysis (e.g., R, MatLab)
- Avoid stating that data files are "available upon request"

eLife Sciences Publications, Ltd is a limited liability non-profit non-stock corporation incorporated in the State of Delaware, USA, with company number 5030732, and is registered in the UK with company number FC030576 and branch number BR015634 at the address 1st Floor, 24 Hills Road, Cambridge CB2 1JP | August 2014

Please indicate the figures or tables for which source data files have been provided:

| |
|---|
| Genetic association analysis: Provided R Scripts and statistical summary Supplementary Files 2 to 4.<br>Fig. 4 A-C, Fig.4-figure supplements 1 and 2, Fig. 5 A-C: All raw data points for respective experiments indicated in /visible through bar graphs and dot plots in respective graphs for directly visible full data transparency. |
